# Frequency and characteristics of delusions and hallucinations in first admitted patients.

**DOI:** 10.1192/j.eurpsy.2023.1844

**Published:** 2023-07-19

**Authors:** M. Abdelkefi, I. Baati, M. Mnif, F. Guermazi, I. Feki, R. Sellami, J. Masmoudi

**Affiliations:** psychiatry A, Hedi Chaker university hospital, sfax, Tunisia

## Abstract

**Introduction:**

Delusions and hallucinations are common in schizophrenia and related psychotic disorders and they are frequently reported at the first admission to psychiatry departments.

**Objectives:**

The study aims to examine the themes and frequency of delusions and hallucinations in first admitted patients.

**Methods:**

Information was collected retrospectively from selected medical files of patients who were admitted for the first time to the department of psychiatry "A" of the university hospital Hedi Chaker, in Sfax, during the years 2020 and 2021.

**Results:**

Ninety patients were included in our study. Their mean age was 34.79 ± 11.4 years, with a sex ratio (M/F) = 1.3. They reached high school in 51.1% of the cases. Half of the patients were smokers, 30% used alcohol and 16.7% used cannabis.

The average age of onset of the disorders was 30.36 years, and the duration of evolution of the illness before hospitalization was 56.54 days. The most common reason for hospitalization was environmental violence (62.5%). The diagnosis of schizophrenia was retained in 32.2% of the cases, and that of bipolar disorder in 23.3% of the cases.

At initial presentation to the ward, 72.2% of patients were found to have delusional beliefs. The most commonly held delusions were delusions of persecution (62.2%), reference (28.9%) bewitchment (27.8%) and grandiosity (26.7%) with changes of behavior in 34.4 % of the patients in response to their delusional beliefs.

Hallucinations reported by 43.3% of the patients were mainly auditory (30%), visual (20%) and 15.6% reported hearing internal voices. Olfactory hallucinations were only reported by 3.3% of the patients.

**Conclusions:**

Delusions of persecution and reference appear to be the main delusional themes in this patient group. Auditory hallucinations were also commonly reported.

A better awareness of clinical presentations of the first admitted patients may aid early identification of the illness and engagement of the patients in the treatment process.

**Disclosure of Interest:**

None Declared

